# Lymph node metastasis-related gene signature shows good performance in predicting prognosis and immune infiltration in cervical cancer

**DOI:** 10.3389/fonc.2023.1190251

**Published:** 2023-06-22

**Authors:** Yilin Guo, Lu Wang, Zhen Xu, Mengqi Li, Wuliang Wang, Yangyang Bai, Xingyue Xu, Rui Li, Hu Zhao

**Affiliations:** ^1^ Department of Gynecology and Obstetrics, The Second Affiliated Hospital of Zhengzhou University, Zhengzhou, China; ^2^ Henan Gynecological Diseases (Gynecology Oncology) Clinical Research Center, Zhengzhou, China; ^3^ Department of Urology, Henan Provincial Hospital of Traditional Chinese Medicine, Zhengzhou, China

**Keywords:** cervical cancer, lymph node metastasis, prognosis, immune infiltration, immunotherapy

## Abstract

**Aims:**

This study aimed to construct a lymph node metastasis-related gene signature to predict prognosis and immune infiltration in patients with cervical cancer.

**Methods:**

Clinical and RNA sequencing data of 193 patients with cervical cancer, which were divided into lymph node metastasis (N1) and non-lymph node metastasis (N0) groups, were acquired from TCGA. Differentially expressed genes (DEGs) between the N1 and N0 groups were detected, and protein-protein interaction combined with LASSO analysis was conducted to further screen lymph node metastasis-related genes. Univariate and multivariate Cox regression analyses were performed to establish a predictive signature. The genetic features, potential biological behavior, and immune infiltration characteristics of the predictive signature were explored. Furthermore, the sensitivity of patients to chemotherapy drugs was estimated based on the predictive signature and the expression of *TEKT2* and *RPGR* was investigated in the cervical cancer tissue samples.

**Results:**

A total of 271 lymph node metastasis-related DEGs, including 100 upregulated and 171 downregulated genes, were identified. Two genes, *TEKT2* and *RPGR*, were associated with lymph node metastasis and prognosis in cervical cancer, and were used to construct a lymph node metastasis-related predictive signature. Based on the predictive signature, patients with cervical cancer were divided into high- and low-risk groups. The high-risk group, characterized by a higher tumor mutation burden and somatic mutation rate, indicated a poor overall survival. The activation of immune infiltration and increased expression of checkpoint genes were observed in the high-risk group, indicating that they might benefit from immunotherapy. Cytarabine, FH535, and procaspase-activating compound-1 were estimated as reasonable chemotherapy options for patients in the high-risk group, whereas two taxanes and five tyrosine kinase inhibitors, including etoposide and vinorelbine, had therapeutic significance for patients in the low-risk group. The expression of *TEKT2* and *RPGR* was significantly downregulated in cervical cancer tissues, especially in metastatic lymph node tissues.

**Discussion:**

The lymph node metastasis-related predictive signature based on *TEKT2* and *RPGR* showed good performance in predicting the survival outcomes of patients with cervical cancer. The risk score of the predictive signature was related to genetic variation and immune infiltration, which could guide immunotherapy and chemotherapy strategies.

## 1 Introduction

Cervical cancer is the fourth most common cancer in women globally, with 341,831 deaths and 604,127 new cases recorded in 2020 (1). Radical hysterectomy and chemoradiotherapy are performed at different stages of cervical cancer (2). However, metastasis and recurrence are the main causes of cancer-related mortality (3). Therefore, it is necessary to identify the key factors that influence the development and metastasis of cervical cancer.

Lymph node metastasis, a multistep process involving complex biological mechanisms, is one of the most crucial prognostic indicators in cervical cancer (4). According to the recently revised 2018 International Federation of Gynecology and Obstetrics staging system for cervical cancer, lymph node status affects the staging of patients with cervical cancer (5). In addition, a study revealed that metastatic lymph nodes exhibited an early stage of immune response, characterized by the infiltration of cytotoxic CD8^+^ T cells, central memory CD4^+^ T cells, and effector memory CD8^+^ T cells, suggesting a correlation between lymph node metastasis and immune infiltration (6). However, the mechanisms underlying lymph node metastasis in cervical cancer remain unclear.

Large-scale genome sequencing studies have identified the molecular signatures of cervical cancer (7). Herein, a comprehensive study of lymph node metastasis in cervical cancer was conducted. A lymph node metastasis-related gene signature was constructed to predict cervical cancer prognosis. We aimed to identify the molecular signature, immune infiltration, and drug sensitivity based on lymph node metastasis to guide the selection of more effective therapeutic strategies for cervical cancer.

## 2 Materials and methods

### 2.1 Data acquisition

The expression profiles and clinical data of 307 cervical squamous cell carcinoma and endocervical adenocarcinoma (CESC) tissue samples were obtained from the Cancer Genome Atlas Genomic Data Commons (TCGA-GDC) Data Portal (https://portal.gdc.cancer.gov/, accessed on November 29, 2022). Perl scripts were used to generate the mRNA, long non-coding RNA (lncRNA) and micro RNA (miRNA). symbol matrix. The Masked Somatic Mutation data were processed through VarScan software and analyzed using “maftools” package in R (version 4.1.2).

### 2.2 Differential analysis of gene expression

Excluding data without lymph node metastases (Nx), patients with CESC (n = 193) were divided into non-lymph node metastasis (N0) and lymph node metastasis (N1) groups. The Kaplan–Meier survival curve was used to compare the differences in overall survival (OS) between the N0 and N1 groups. Differentially expressed mRNA, lncRNA, and miRNA were analyzed using the “limma” package with the criteria of an absolute log2-fold-change (FC) > 0.5 and *P* value <0.05. Heatmaps and volcano plots were constructed using the “ggplot2” and “pheatmap” packages, respectively, to display the expression of differentially expressed genes (DEGs), which were defined as lymph node metastasis-related genes.

### 2.3 Construction of a ceRNA regulatory network

To investigate the potential existence of DEGs involved in a competing endogenous RNA (ceRNA) network mediated by differentially expressed lncRNA and miRNA, we conducted miRanda (http://www.microrna.org/), miRWalk (http://129.206.7.150/), and Targetscan (http://www.targetscan.org/) analyses to predict miRNA-mRNA interactions, while StarBase (https://starbase.sysu.edu.cn/) was used to predict lncRNA-miRNA interactions. The resulting interactions were then visualized using Cytoscape software (version 3.9.1).

### 2.4 Functional enrichment analysis

To investigate the different biological processes of lymph node metastasis-related DEGs, Gene Ontology (GO) and Kyoto Encyclopedia of Genes and Genomes (KEGG) enrichment analyses were performed using the “org.Hs.eg.db,” “clusterProfiler,” “enrichplot,” and “ggplot2” packages, with the *P* value threshold set at <0.05. Gene set variation analysis (GSVA) was performed using the “GSVA” package in R to investigate the common activated/suppressed biological process. The gene sets of the “c2.cp.kegg.v7.4. symbols.gmt,” and “c5.go.v7.4. symbols.gmt” were downloaded from the Molecular Signatures Database (8). Statistical significance was set at *P <*0.05.

### 2.5 Protein-protein interaction network construction

To analyze the protein interactions among lymph node metastasis-related DEGs, the Search Tool for the Retrieval of Interacting Genes/Proteins Database (http://string-db.org) was used to construct a PPI network with a reliable filtering condition (score >0.3). The node gene score was determined using the “cytoHubba” plugin of the Cytoscape software (version 3.9.1), and the top 30 node genes scored by the degree algorithm were defined as hub genes.

### 2.6 Predictive signature construction

The least absolute shrinkage and selection operator (LASSO) binomial logistic regression analysis was used to regress the high-dimensional prediction factors, as previously reported (9). Univariate and multivariate Cox regression analyses were conducted to further screen for lymph node metastasis-related genes and establish a predictive signature. The risk score of the predictive signature was calculated as follows: Risk score = (exp-Gene_1_*coef-Gene_1_) + (exp-Gene_2_*coef-Gene_2_) +……+ (exp-Gene_i_*coef-Gene_i_). The patients were divided into high- and low-risk groups based on their median risk scores. The Kaplan–Meier survival curve was used to evaluate survival differences between the two groups. Time-dependent receiver operating characteristic (ROC) curves were used to evaluate survival predictions, and the “timeROC” package was used to calculate the area under the ROC curve (AUC) to assess the accuracy of the prognostic predictive signature. Univariate and multivariate Cox regression analyses were conducted to compare the hazard ratio of the predictive signature with characteristic clinical features of cervical cancer.

### 2.7 Nomogram construction and validation

To determine the effective clinical application of the predictive signature, a nomogram for predicting the 1-, 3- and 5-year survival rate of patients with CESC was constructed by combining the results of the risk score through the “rms” package in R. The predictive accuracy and discrimination ability of the nomogram were assessed using a calibration plot and the c-index. The sensitivity and specificity of the nomogram were evaluated using the AUC.

### 2.8 Copy number variation and somatic alteration data analysis

Compliant datasets were subjected to CNV analysis. CNVs of the genes screened through LASSO binomial logistic regression analysis in patients with CESC were analyzed. The somatic mutation data of CESC between the high- and low-risk groups, which were classified using the predictive signature, were obtained using the “maftools” package. The tumor mutation burden (TMB), which has been proposed as an immunotherapy efficacy predictor, of each patient with CESC was calculated using the “tmb ()” function of the “maftools” package in R. The correlation between the TMB and risk score was analyzed using the Wilcoxon rank-sum test.

### 2.9 Immune cell infiltration and immunotherapy response analyses

The CIBERSORT deconvolution algorithm, which is based on the principles of linear support vector regression, was used to obtain 22 immune cell infiltrations in CESC samples according to the known reference set LM22 with *P* values <0.05 (10). The stromal content (StromalScore), immune infiltration (ImmuneScore), and combined score (ESTIMATEScore) of each CESC sample were calculated by applying the ESTIMATE algorithm. The single-sample gene set enrichment analysis (ssGSEA) algorithm was used in the “GSVA” package to evaluate the relative abundance of infiltrated immune cells. Immune cell type-related marker genes were obtained from Bindea et al (11). The association between the risk score of the predictive signature and the expression of immune checkpoint genes, such as programmed cell death protein ligand 1 (*PD-L1*), cytotoxic T-lymphocyte-associated protein 4 (*CTLA-4*), and human leukocyte antigen (*HLA*), was assessed.

### 2.10 Drug sensitivity analysis

The Genomics of Drug Sensitivity in Cancer (GDSC) (https://www.cancerrxgene.org/ ) database was used to obtain tumor drug response data and genomic drug sensitivity markers (12). The half-maximal inhibitory concentration (IC50) values of common drugs between the high-risk and low-risk groups of patients with CESC, which were grouped using the predictive signature, were estimated using the pRRophetic algorithm in R.

### 2.11 Human tissue samples and cell line

Seven cervical cancer (three cases with lymph node metastasis and four cases without lymph node metastasis) and three normal cervical tissue samples were collected from patients with cervical cancer who underwent hysterectomy before chemotherapy and radiotherapy. The median age of the seven cases was 48 years (range, 32–58). Six cases were HPV positive, and one case was HPV negative. Five cases were squamous cell carcinoma, and two cases was adenocarcinoma. All tissues were stored at −80°C for RNA extraction. The studies involving human participants was approved by the Ethics Committee of the Second Affiliated Hospital of Zhengzhou University (2021040).

The human cervical cancer cell line, SiHa, was obtained from Procell Life Science & Technology Co., Ltd. (Wuhan, China) and cultured in Dulbecco’s modified Eagle’s medium (DMEM) supplemented with 10% fetal bovine serum (Gibco, Grand Island, USA). SiHa cells were maintained in a 5% CO_2_ and 95% air incubator.

### 2.12 RNA extraction and quantitative real-time PCR

Total RNA was extracted from tissues and cells using TRIzol reagent (Invitrogen, Carlsbad, CA, USA). The purity and quantity of RNA was determined by measuring the absorbance at 260/280 nm using a SmartSpec Plus Spectrophotometer (Bio-Rad Laboratories, Inc., Hercules, CA, USA). Reverse transcription was carried out using the ReverTra Ace qPCR RT Kit (TOYOBO Life Science, Shanghai, China), according to the manufacturer’s instructions. qRT-PCR was performed using the Bestar SYBR Green qPCR Master Mix (TOYOBO) on a Bio-Rad S1000 system. For quantitative RT-PCR, *GAPDH* was used as an endogenous control, using the 2−^ΔΔCT^ method. All primer sequences are listed in **Supplementary Table 1**.

### 2.13 Cellular transfection

Small interfering RNAs (siRNA) directed against *TEKT2* (si-*TEKT2*) and negative control RNAs (si-NC) were synthesized by GeneCreate Bioengineering Co., Ltd. (Wuhan, P.R. China). Transfections were performed using Lipofectamine 2000 (Invitrogen). The siRNA sequences are listed in **Supplementary Table 2**.

### 2.14 Colony formation assay

For the colony formation assay, 1.5 × 10^3^ SiHa cells were maintained in 6-well culture plates. After 14 d of incubation, the colonies were fixed with methanol and stained with a crystal violet solution. Colonies containing more than 50 cells were counted.

### 2.15 Wound healing assay

Approximately 5 × 10^5^ SiHa cells per well were seeded in 6-well culture plates. After 24 h of incubation, the center of each well was scraped with a 200 μl sterile pipette tip. The cells were washed three times with phosphate-buffered saline, and fresh DMEM was added. The wound was imaged at three time points after 0, 24, and 48 h.

### 2.16 Apoptosis analysis

Cell apoptosis was analyzed using an Annexin V-FITC Apoptosis Detection Kit (Solarbio, Beijing, China), according to the manufacturer’s recommendations, using a FACSCalibur flow cytometer (BD Biosciences, USA).

### 2.17 Statistical analysis

All statistical analyses and illustrations were performed using R software (version 4.1.2, https://www.r-project.org/). All *P* values were two-sided, with *P <*0.05 defined as statistically significant. Wilcoxon’s test was used to compare differences between two groups, and one-way analysis of variance and Kruskal–Wallis tests were used as parametric and non-parametric methods, respectively, for comparisons among three or more groups.

## 3 Results

### 3.1 Identification of DEGs associated with lymph node metastasis

**Figure 1:**
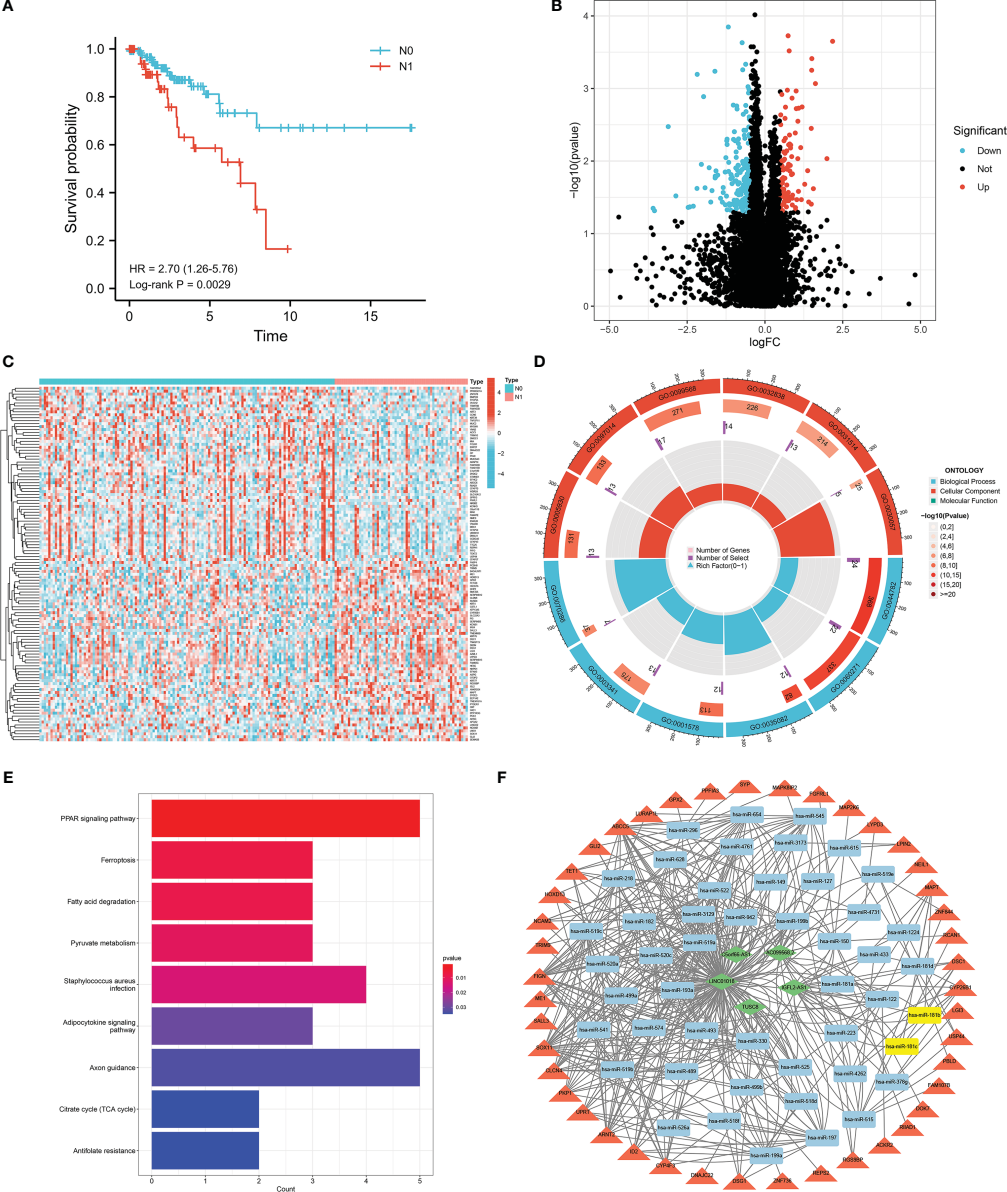
Identification of DEGs related to lymph node metastasis. **(A)** Survival analyses of lymph node metastasis and non-lymph node metastasis in TCGA-CESC database. **(B)** Volcano plot of DEGs related to lymph node metastasis. **(C)** The heatmap of 60 up-regulated and 60 down-regulated DEGs between cervical cancer patients with and without lymph node metastasis. **(D)** Gene Ontology (GO) functional analysis of 271 DEGs, including biological process (BP), and cellular component (CC). **(E)** Kyoto Encyclopedia of Genes and Genomes (KEGG) pathways of 271 DEGs related to lymph node metastasis. **(F)** lncRNA-miRNA-mRNA ceRNA network in cervical cancer with lymph node metastasis. Diamond, round rectangle and triangle represent differentially expressed lncRNA, miRNA, and DEGs, respectively. Yellow nodes indicate differentially expressed miRNA.

We downloaded the expression profiles and clinical data of 307 patients with CESC from the TCGA-CESC database. Excluding data without lymph node metastases (Nx), 193 CESC patients were enrolled for subsequent analysis. The baseline data, including clinical and pathological characteristics, are presented in **Table 1**. The median patient age was 46 years (range, 29–72 years). Kaplan–Meier survival curves showed that the OS rate of patients with CESC in the lymph node metastasis (N1) group was significantly lower than that in the non-lymph node metastasis (N0) group (*P* = 0.0029, **Figure 1A**), indicating that lymph node metastasis was associated with survival of patients with CESC and lymph node metastasis-related genes might serve as prognostic biomarkers.

**Table 1:** Summary of the clinical characteristics of 193 CESC patients.

Features	N0 groups (n = 133)	N1 groups (n = 60)	Total (n = 193)	*P* value
Age (years)				
<=35	26	15	41	0.424
>35, <=65	94	42	136	
>=65	13	3	16	
Grade				
G1	13	1	14	0.168
G2	58	28	86	
G3	56	28	84	
Gx	6	3	9	
Stage				
I	99	29	128	<0.001
II	29	8	37	
III	3	19	22	
IV	2	2	4	
Unknown	0	2	2	
T				
T1	98	32	130	0.017
T2	31	20	51	
T3	2	6	8	
T4	1	1	2	
Unknown	1	1	2	
M				
M0	80	27	107	0.128
M1	2	2	4	
Unknown	51	31	82	
Pathological type				
SCC	104	51	155	0.545
AC	26	8	34	
ACS	3	1	4	
Race				
White	98	39	137	0.639
Black or african american	10	8	18	
Asian	12	6	18	
American indian	1	0	1	
Native hawaiia	1	0	1	
Unknown	11	7	18	
Vital status				
Dead	16	17	33	0.005
Alive	117	43	160	

FIGO, Federation of International of Gynecologists and Obstetricians; SCC, Squamous cell carcinoma; AC, Adenocarcinoma; ASC, Adenosquamous carcinoma.

A total of 271 lymph node metastasis-related DEGs, including 100 upregulated and 171 downregulated genes, were obtained (**Figure 1B**). The heatmap showed 60 upregulated and 60 downregulated genes between the N0 and N1 groups of patients with CESC (**Figure 1C**). To investigate the different biological processes of the DEGs, GO and KEGG pathway enrichment analyses were performed. The main biological functions were the axoneme, cytoplasmic region, cilium organization, and cilium assembly (**Figure 1D**). DEGs were mainly enriched in the PPAR signaling pathway, ferroptosis, fatty acid degradation, and pyruvate metabolism (**Figure 1E**).

To find out whether these DEGs existing competing endogenous regulating network, a lncRNA-miRNA-mRNA ceRNA network of cervical cancer with lymph node metastasis was constructed. A total of 5 differentially expressed lncRNAs and 48 miRNAs were paired into 228 lncRNA-miRNA interactions, whereas 48 miRNAs and 43 DGEs were matched to form 228 pairs of miRNA-mRNA interactions (**Figure 1F**).

### 3.2 PPI network construction and lymph node metastasis-related hub gene screening

**Figure 2:**
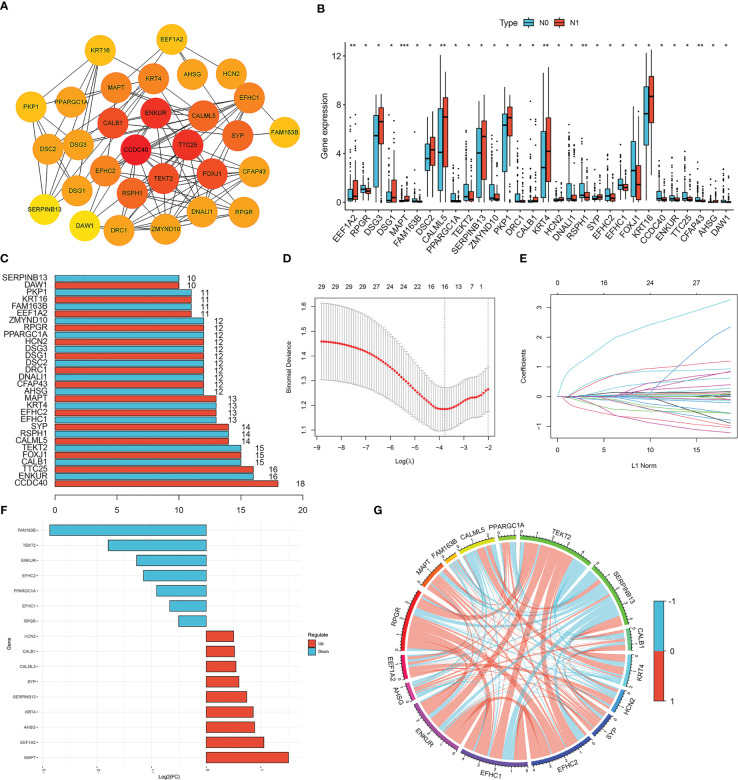
Screening of lymph node metastasis-related hub genes. **(A)** Protein-protein interaction (PPI) network of DEGs related to lymph node metastasis. **(B)** The expression of 30 hub genes between cervical cancer patients with and without lymph node metastasis. Red represents lymph node metastasis, and blue represents non-lymph node metastasis. The asterisks represented the statistical p value (*P < 0.05; **P < 0.01; ***P < 0.001). **(C)** The number of genes associated with other hub genes. **(D, E)** LASSO binomial logistic regression analysis for further screening of hub genes. **(F)** The expression of 16 hub genes between cervical cancer patients with and without lymph node metastasis. **(G)** Correlation analysis of 16 hub genes in cervical cancer. Blue represents negative correlation, and red represents positive correlation.

We conducted further interactive network analysis of the lymph node metastasis-related DEGs and found that there were 30 hub genes scored by the degree algorithm (**Figures 2A, C**). The expression of the 30 hub genes between the N0 and N1 groups is shown in **Figure 2B**. LASSO binomial logistic regression analysis was performed to further screen hub genes related to lymph node metastasis. The analysis results revealed 16 hub genes (**Figure 2D, E**). The expression of *FAM163B*, *TEKT2*, *EFHC1, ENKUR, PPARGC1A, EFHC2*, and *RPGR* was lower in the N1 group than in the N0 group (**Figure 2F**). In contrast, *EEF1A2, MAPT, CALML5, SERPINB13, CALB1, KRT4, HCN2, SYP*, and *AHSG* expression was upregulated in the N1 group (**Figure 2F**). Additionally, a correlation was found among the 16 hub genes (**Figure 2G**).

### 3.3 Establishment of the prediction signature and nomogram

**Figure 3:**
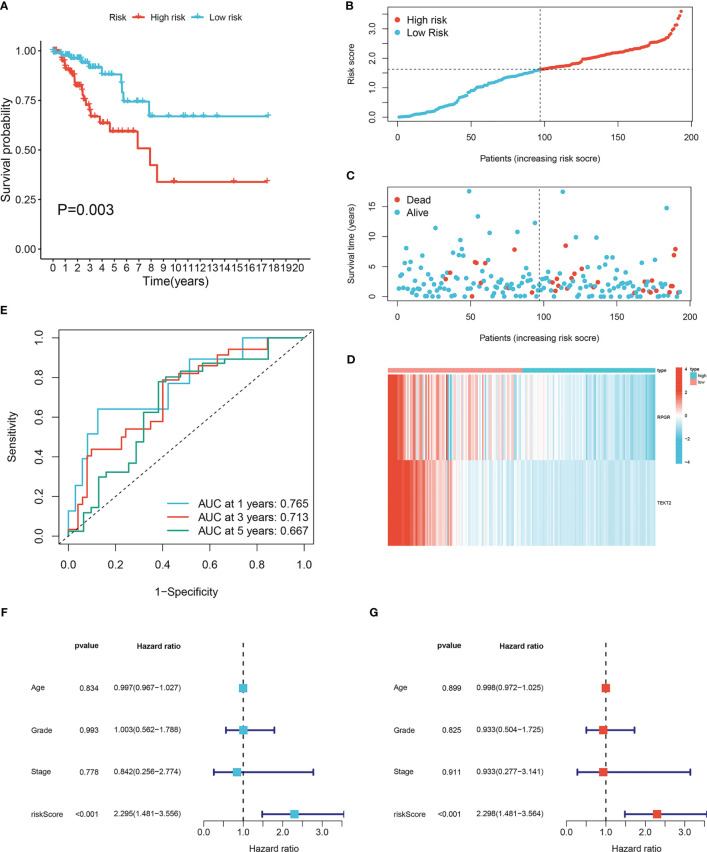
Construction lymph node metastasis-related predictive signature. **(A)** Kaplan-Meier survival curves of high- and low-risk groups in cervical cancer. **(B)** Cervical cancer patients were divided into high- and low-risk groups according to the median of the risk score. **(C)** Risk score and survival status for each cervical cancer patient. **(D)** The heatmap of the expression levels of *RPGR* and *TEKT2* between high- and low-risk groups. **(E)** ROC curve for 1-, 3-, and 5-years survival of the predictive signature. **(F)** Univariate Cox analysis of the clinicopathological features and risk predictive signature. **(G)** Multivariate Cox analysis of the clinicopathological features and risk predictive signature.

To investigate the prognostic value of the 16 lymph node metastasis-related hub genes, univariate and multivariate Cox regression analyses were performed to calculate the hazard ratio and establish a lymph node metastasis-related predictive signature. Risk score = (−0.7983 × expression level of *RPGR*) + (−0.6974 × expression level of *TEKT2*). A total of 193 patients with CESC were divided into high- and low-risk groups according to the median risk score, and the risk score curve was plotted (**Figure 3B**). Survival curves demonstrated that the survival rate in the high-risk group was notably lower than that in the low-risk group (*P*=0.003; **Figures 3A, C**). The expression heatmap showed that *RPGR* and *TEKT2* was downregulated in the high-risk group (**Figure 3D**). The ROC curve was used to predict prognosis at 1, 3, and 5 years, and the prediction efficiency was satisfactory (1-year AUC = 0.765; 3-year AUC = 0.713; 5-year AUC = 0.667; **Figure 3E**). Univariate and multivariate Cox analyses showed that the predictive signature was an independent prognostic factor in patients with CESC (*P*<0.001; **Figures 3F, G**). Furthermore, a nomogram was constructed to predict the 1-, 3-, and 5-year survival rates of patients with CESC (**Supplementary Figure 1A**), and the calibration plot confirmed its effective predictive performance (**Supplementary Figure 1B–D**). The c-index was 0.705, and the AUCs for the 1-, 3-, and 5-year survival curves of patients with CESC were 0.760, 0.718, and 0.687, respectively (**Supplementary Figure 1E**).

### 3.4 Correlations between prediction signature and clinicopathological characteristics

**Figure 4:**
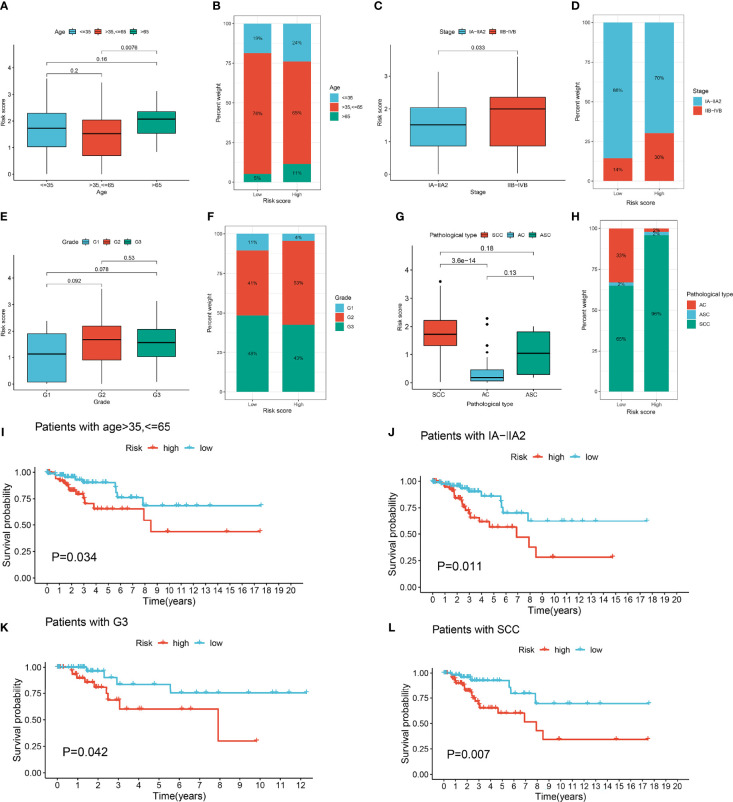
Clinical correlation analysis of lymph node metastasis-related prediction signature in cervical cancer. **(A)** Boxplots of risk score in different age groups. **(B)** The proportion of different age groups between high- and low-risk groups. **(C)** Boxplots of risk score in different stage groups. **(D)** The proportion of different stage groups between high- and low-risk groups. **(E)** Boxplots of risk score in different grade groups. **(F)** The proportion of different grade groups between high- and low-risk groups. **(G)** Boxplots of risk score in different pathological type groups. **(H)** The proportion of different pathological type groups between high- and low-risk groups. **(I)** Kaplan-Meier survival curves of age >35, <=65 in high- and low-risk groups. **(J)** Kaplan-Meier survival curves of IA-IIA2 in high- and low-risk groups. **(K)** Kaplan-Meier survival curves of G3 in high- and low-risk groups. **(L)** Kaplan-Meier survival curves of pathological type squamous cell carcinoma (SCC) in high- and low-risk groups.

We investigated the correlation between the lymph node metastasis-related prediction signature and clinicopathological characteristics of cervical cancer. Stratified analysis indicated significant differences in the risk score of the prediction signature among groups divided by age and stage and pathological type, but not by grade (**Figures 4A, C, E, G**). The proportion of patients with CESC in the high-risk group with clinicopathological characteristics including age ≤35 years, age >65 years, stage IIB-IVB, and stage G2 and pathological type squamous cell carcinoma (SCC) was higher than those in the low-risk group (**Figures 4B, D, F, H**). Kaplan–Meier survival curves showed that the patients with CESC in the low-risk group with clinicopathological characteristics including age >35 years, age ≤65 years, and stage IA-IIA, G1, and G3, and pathological type SCC had better prognosis than those in the high-risk group (*P* < 0.05; **Figure 4I–L**). Conversely, there was no significant difference in prognosis between the high- and low-risk groups of patients with CESC characterized by age ≤35 years, age >65 years, and stage IIB-IVB and G2 and pathological type adenocarcinoma (AC), adenosquamous carcinoma (ACS).

### 3.5 Influence of prediction signature on genomic changes and biological function

**Figure 5:**
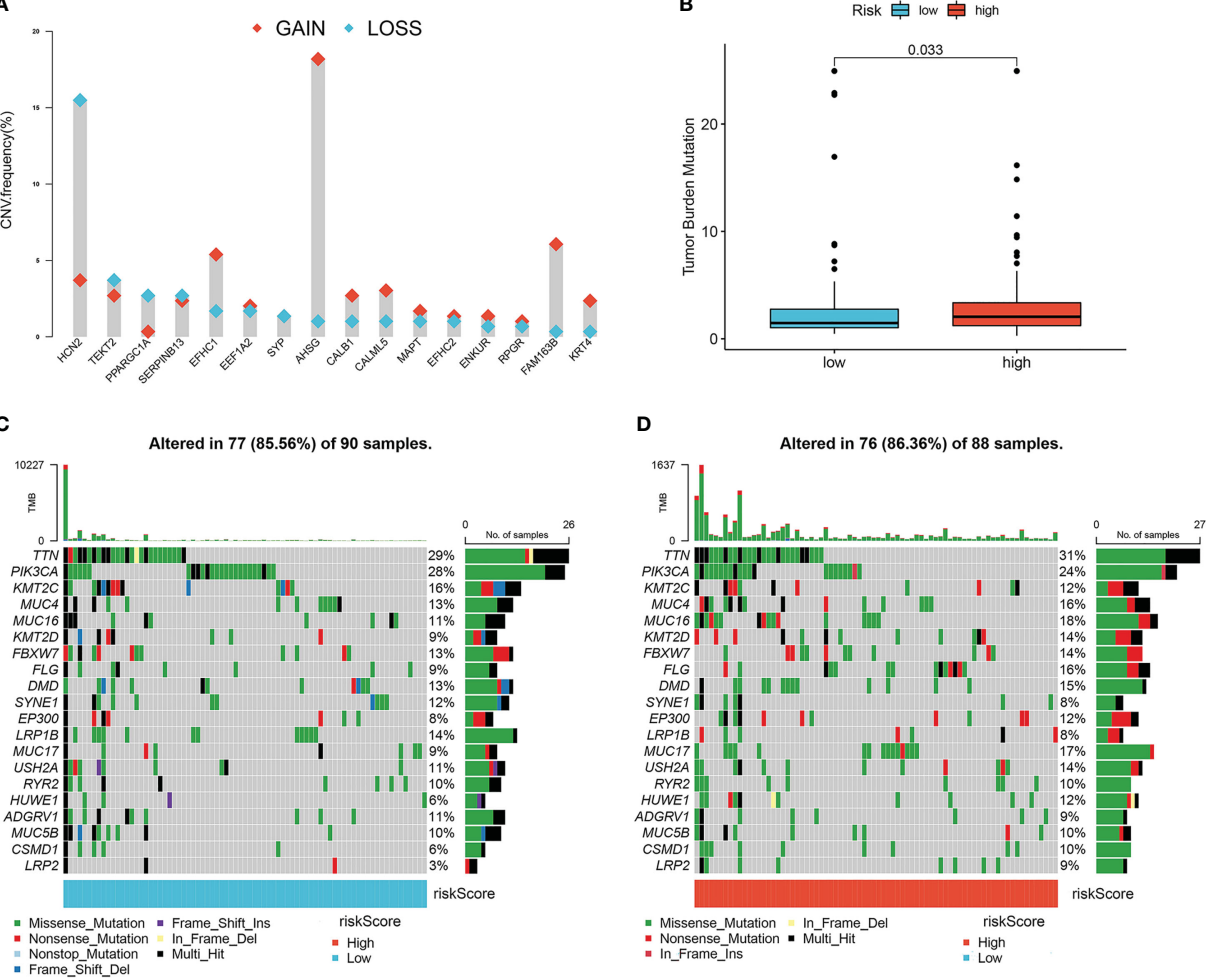
Characteristics of tumor somatic mutations in lymph node metastasis-related predictive signature. **(A)** The CNV variation frequency of 16 hub genes. Red represents an increase in copy number, and blue represents loss of copy number. **(B)** Boxplots of tumor mutation burden between high- and low-risk groups. **(C)** The mutation frequency of low-risk groups of cervical cancer patients. **(D)** The mutation frequency of high-risk groups of cervical cancer patients.

To detect the genetic features and potential biological behavior of the lymph node metastasis-related prediction signature, we first analyzed the CNVs of the 16 lymph node metastasis-related hub genes. Analysis revealed that CNV alterations in the 16 hub genes were prevalent, with higher probabilities of CNV deletions in *HCN2*, *TEKT2*, and *PPARGC1A* and CNV amplifications in *AHSG*, *FAM163B*, *EFHC1*, and *RPGR* (**Figure 5A**).

We then compared the TMB and somatic variations between the high- and low-risk groups. The TMB score in the high-risk group was higher than that in the low-risk group (*P*=0.033; **Figure 5B**), which indicated that the high-risk group was more likely to benefit from PD-1 inhibitors. Missense mutations accounted for most mutations, and *TTN* was the most common mutated gene (**Figures 5C, D**). The high-risk group had a higher somatic mutation rate than the low-risk group (86.63% vs. 85.56%).

Finally, we conducted GSVA analysis between the high- and low-risk groups. The main biological roles were wide pore channel activity, desmosome organization, and regulation of epidermis development in the high-risk group and extracellular transport and protein transport along microtubules in the low-risk group (**Supplementary Figure 2A**). Toll-like receptor, VEGF, P53, T cell receptor, B cell receptor, JAK STAT, TGF-β, and natural killer cell-mediated cytotoxicity signaling pathways were positively correlated with the risk score of the prediction signature (**Supplementary Figures 2B, C**).

### 3.6 Influence of prediction signature on immune characteristics

**Figure 6:**
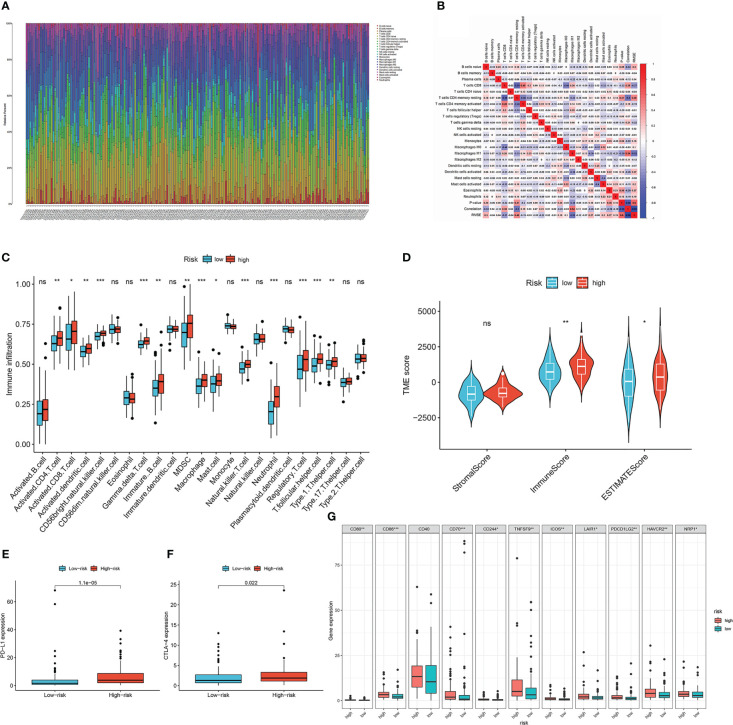
Characteristics of tumor microenvironment in lymph node metastasis-related predictive signature. **(A)** The panorama of immune cell infiltration in TME of cervical cancer. **(B)** Correlation analysis of 22 types of immune cells in cervical cancer. Blue represents negative correlation, and red represents positive correlation. **(C)** The infiltration of immune cell types between the high- and low-risk groups by ssGSEA analysis. The asterisks represented the statistical p value (*P < 0.05; **P < 0.01; ***P < 0.001). **(D)** Immunological score versus stromal score in high- and low-risk groups by ESTIMATE analysis. The asterisks represented the statistical p value (*P < 0.05; **P < 0.01). **(E)** Boxplots of the expression of *PD-L1* in high- and low-risk groups. **(F)** Boxplots of the expression of *CTLA-4* in high- and low-risk groups. **(G)** Differential expression analysis of other checkpoint genes in high- and low-risk groups. The asterisks represented the statistical p value (*P < 0.05; **P < 0.01; ***P < 0.001).

To investigate the relationship between the lymph node metastasis-related prediction signature and immune cell infiltration in the CESC microenvironment, we calculated the proportions of 22 immune cell types in 193 samples using the CIBERSORT algorithm. The panorama of immune cell infiltration in the CESC microenvironment is shown in **Figure 6A**, and the correlations between the 22 immune cell types is shown in **Figure 6B**. Resting memory CD4 T cells showed the highest negative correlation with CD8 T cells (cor =−0.59), followed by activated memory CD4 T cells (cor = −0.46). Activated memory CD4 T cells displayed the highest positive correlation with CD8 T cells (cor = 0.49). We then compared the infiltration of immune cell types between the high- and low-risk groups using ssGSEA (**Figure 6C**). Immune infiltration of activated CD8 T cells, activated CD4 T cells, activated dendritic cells, monocytes, regulatory T cells, and type 1 T helper cells was higher in the high-risk group than in the low-risk group (*P <*0.05). Using ssGSEA, we also compared the relationship between *TEKT2* and *RPGR* and differentially distributed immune cells (**Supplementary Figure 3A**). *TEKT2* and *RPGR* expression levels were negatively correlated with almost all immune cells, including gamma delta T cells, macrophages, natural killer T cells, regulatory T cells, T follicular helper cells, and type 1 T helper cells (*P <*0.05). Furthermore, the ESTIMATE algorithm was applied to determine the differences in the stromal content, immune infiltration, and combined score between the high- and low-risk groups (**Figure 6D**). The results showed that the ImmuneScore and ESTIMATEScore in the high-risk group were higher than those in the low-risk group (*P <*0.05).

To predict the immunotherapy response of patients with CESC, we detected the differential expression of checkpoint genes in the low- and high-risk groups (**Figures 6E–G**). Patients in the high-risk group showed high expression of *PD-L1*, *CTLA-4*, *CD80*, *CD86*, *CD70*, *CD244*, *TNFSF9*, *ICOS*, *LAIR1*, *PDCD1LG2*, *HAVCR2*, and *NRP1* (*P <*0.05). *TEKT2*, *RPGR*, and the risk score of the lymph node metastasis-related prediction signature were significantly correlated with checkpoint genes (**Supplementary Figure 3B**). The expression of *TEKT2* and *RPGR* was negatively correlated with that of *PD-L1* and *CTLA-4*, whereas the risk score was positively correlated with the expression of *PD-L1* and *CTLA-4*, which was consistent with the results shown in **Figures 6E–G**. Moreover, we analyzed the differential expression of human leukocyte antigen (HLA) family genes in the high- and low-risk groups (**Supplementary Figure 3C**). Excluding *HLA-E* and *HLA-DQA2*, there were no statistically significant differences between the two groups (*P >*0.05).

### 3.7 Prediction signature sensitivity-based analysis of chemotherapy drugs

**Figure 7:**
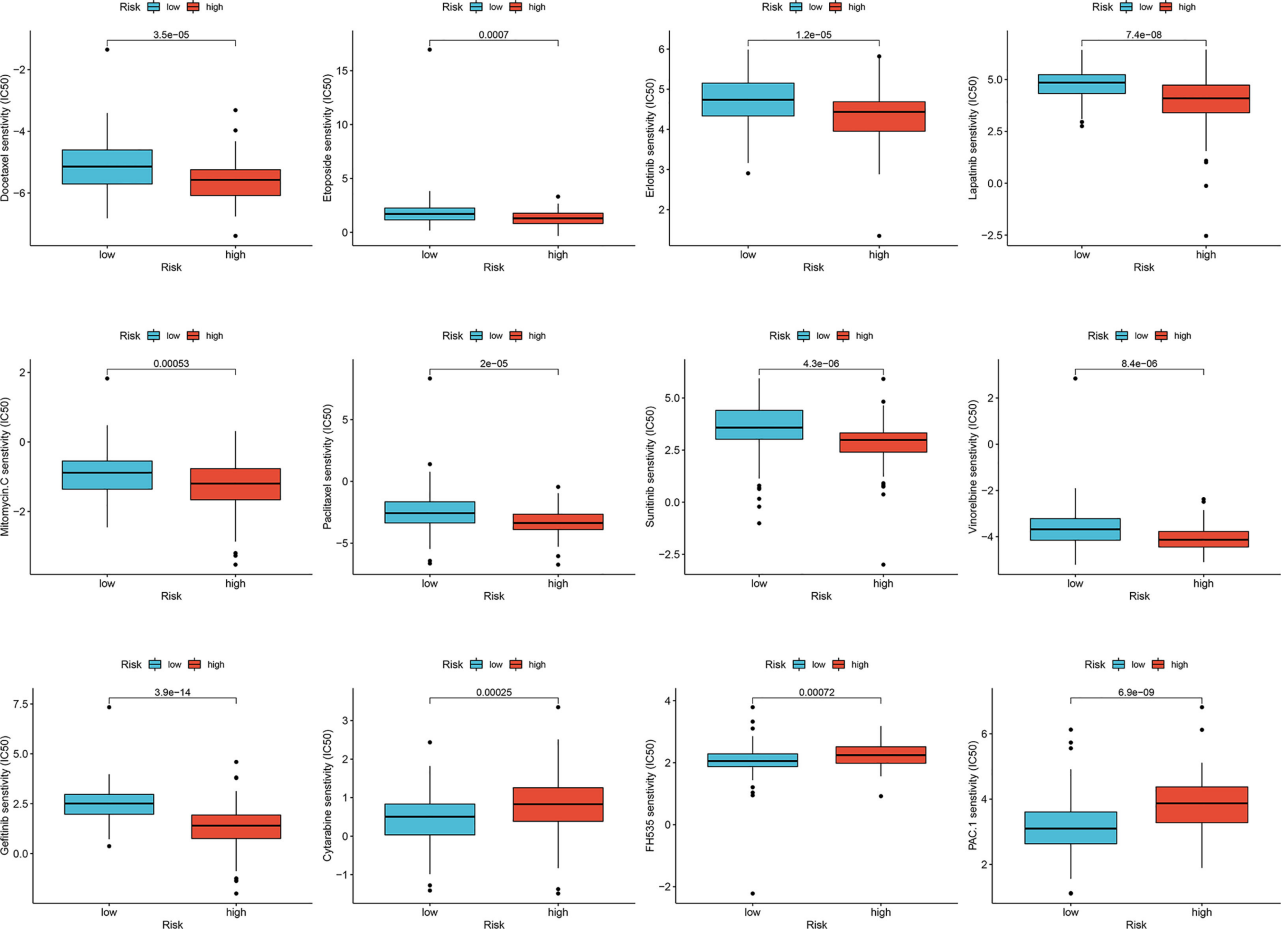
Sensitivity of drugs in lymph node metastasis-related predictive signature. Differences in IC50 values for high- and low-risk groups in cervical cancer.

Considering that chemotherapy is an adjuvant treatment for CESC, we accessed the GDSC database to evaluate susceptibility to common antineoplastic agents in the high- and low-risk groups. We calculated the IC50 values of 138 chemotherapy drugs and analyzed the relationship between the risk groups and IC50 values. The IC50 estimates of docetaxel, paclitaxel, etoposide, erlotinib, lapatinib, sunitinib, gefitinib, and vinorelbine in the low-risk group were significantly higher than those in the high-risk group, whereas the IC50 estimates of cytarabine, FH535, and procaspase-activating compound-1 (PAC-1) in high-risk group were significantly higher than those in the low-risk group (**Figure 7**). Collectively, these results proved that the risk score of the lymph node metastasis-related prediction signature had good reliability.

### 3.8 Expression and function of *TEKT2* and *RPGR* in cervical cancer

**Figure 8:**
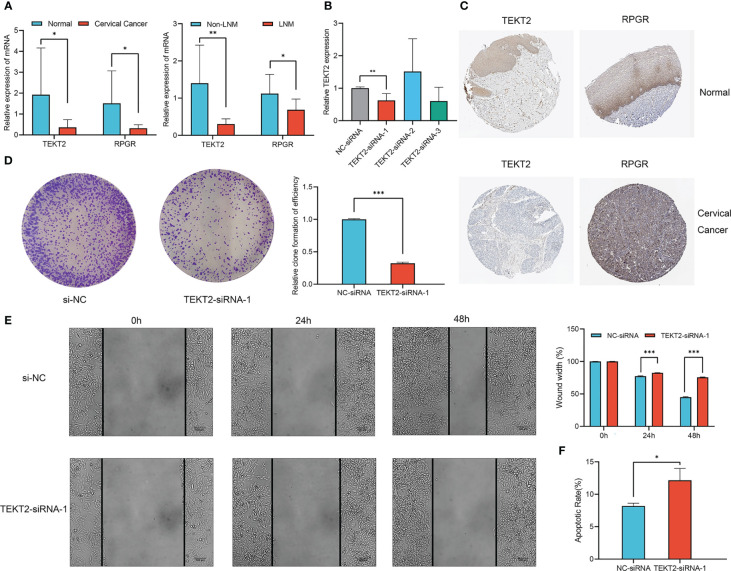
Expression and function of *TEKT2* and *RPGR* in Cervical Cancer. **(A)** The mRNA expression of *TEKT2* and *RPGR* in cervical cancer tissues versus normal cervical tissues, and cervical cancer tissues with lymph node metastatic versus cervical cancer tissues without lymph node metastatic. The asterisks represented the statistical p value (*P < 0.05; **P < 0.01). **(B)** The transfection knockdown efficiency of *TEKT2* in the SiHa cell line using siRNA. The asterisks represented the statistical p value (**P < 0.01). **(C)** The protein expression of *TEKT2* and *RPGR* in cervical cancer tissues and normal cervical tissues in Human Protein Atlas data. **(D)** Proliferation of SiHa cells was detected by colony formation after *TEKT2* knockdown. The asterisks represented the statistical p value (***P < 0.001). **(E)** Migration of SiHa cells was detected by wound healing assay after *TEKT2* knockdown. The asterisks represented the statistical p value (***P < 0.001). **(F)** Apoptosis of SiHa cells after *TEKT2* knockdown. The asterisks represented the statistical p value (*P < 0.05).

To clarify the role of TEKT2 and RPGR in cervical cancer, we investigated *TEKT2* and *RPGR* expression in seven cervical cancer tissues and three normal cervical tissues. RT-qPCR results revealed that the mRNA expression of *TEKT2* and *RPGR* was significantly downregulated in tumor samples, especially in metastatic lymph node samples (**Figure 8A**). Analysis of the Human Protein Atlas data revealed that TEKT2 and RPGR protein levels were also downregulated in cervical cancer samples (**Figure 8C**).

To explore the function of *TEKT2* in cervical cancer, *TEKT2* was efficiently knocked down in the SiHa cell line using siRNA. RT-qPCR validated the transfection knockdown efficiency (**Figure 8B**). The colony formation assay revealed that the proliferation of SiHa cells decreased after *TEKT2* knockdown (**Figure 8D**). Moreover, *TEKT2* knockdown remarkably reduced the migration and increased the apoptosis of SiHa cells (**Figure 8E, F**).

## 4 Discussion

Cervical cancer is a common malignancy that affects women’s health. In developing countries, especially India and China, cervical cancer has higher incidence and mortality rates (13). Human papillomavirus (HPV) is a critical inducing agent in the development of cervical cancer and is detected in more than 90% of cases (14, 15). Early stage cervical cancer is successfully managed with radical hysterectomy with pelvic lymphadenectomy, and more advanced-stage tumors are treated with chemoradiotherapy (16). However, few effective treatment options exist for recurrent or metastatic cervical cancer (17). Recently, sequencing analysis of a large number of clinical tissues has improved our understanding of the molecular signatures of cervical cancer (18). Thus, exploring new therapeutic targets and biomarkers using bioinformatic analyses has enabled precise treatment options, which could significantly improve patient outcomes.

Lymph node metastasis is common in cervical cancer and has prognostic implications for patients (19). Recent studies suggest that lymphatic vessels do not merely provide channels for tumor spread, but also promote tumor cell recruitment to lymph nodes, immune modulation, and cancer stem cell survival (20). In addition, epithelial-mesenchymal transition (EMT) is considered a biomarker of tumor cell metastasis and is closely related to lymph node metastasis (21). However, a detailed understanding of lymph node metastasis in cervical cancer is lacking.

Here, we downloaded the expression profiles and clinical data of 193 patients with CESC from the TCGA-CESC database. A total of 271 lymph node metastasis-related DEGs were identified, including 100 upregulated and 171 downregulated genes. Through interactive network and LASSO binomial logistic regression analyses, we identified 16 hub genes. Two genes, *TEKT2* and *RPGR*, were screened from these 16 hub genes using univariate and multivariate Cox regression analyses to construct the lymph node metastasis-related prognostic gene signature. By calculating the risk score for each patient, we divided them into high- and low-risk groups. The signature demonstrated good performance in predicting the survival outcomes of patients with cervical cancer.

TEKT2 and other members of the tektin protein family are critical for cytoskeleton formation (22). Several studies have revealed that TEKT2 is associated with spermatogenesis and sperm flagellar movement (23). The expression of *TEKT2* was downregulated in the lymph node metastasis group, suggesting that it is a protective factor in cervical cancer, which is consistent with the results of Yang et al. (24). *RPGR* encodes the GTPase regulator in retinitis pigmentosa, and its mutation is linked to severe multisystem diseases with strong retinal involvement of photoreceptor neurons (25). In addition, *RPGR* is one of the top driver oncogenes in breast cancer (26). Lin et al. (27) identified the regulatory competing endogenous RNAs of *RPGR* in nasopharyngeal carcinoma. However, the role of *RPGR* in cervical cancer remains unclear and requires further investigation. The expression of *TEKT2* and *RPGR* was significantly downregulated in cervical cancer tissues, especially in lymph node metastatic tissues. *TEKT2* knockdown inhibited cervical cancer cell proliferation and migration but promoted apoptosis. However, the biological function of *TEKT2* in SiHa cells was inconsistent with the tissue expression analysis results, and thus requires further study.

*TEKT2* and *RPGR* have been found to be associated with lymph node metastasis in cervical cancer. Interestingly, several studies have constructed different models for lung adenocarcinoma (28), bladder cancer (29), and thyroid cancer (30) to predict lymph node metastasis or prognosis. To better understand the genetic features and potential biological behavior of the lymph node metastasis-related predictive signature, we compared the TMB between high- and low-risk groups and performed GSVA analysis. The TMB score in the high-risk group was higher than that in the low-risk group. Higher TMB results in more neoantigens, increasing the chances of T cell recognition, and clinically correlates with better immune checkpoint inhibitor (ICI) efficacy (31). GSVA analysis revealed that T and B cell receptors were positively correlated with the risk score, which indicated that the prediction signature had a strong relationship with immunity.

Cervical cancer is significantly associated with persistent HPV infection. Lymph nodes are common sites of cervical cancer metastasis, and tumor cells can interact with the host immune system by controlling the infiltration and reactivity of immune cells (32). This indicates that lymph node metastasis may be closely associated with immune cell infiltration. Therefore, we compared the infiltration of immune cells between the high- and low-risk groups and found that the abundance of CD8 + T cells, activated CD4 + T cells, and activated dendritic cells was significantly higher in the high-risk group than in the low-risk group. Similarly, our findings illustrated that the ESTIMATEScore, which is defined as the percentage of tumor cells in the tumor microenvironment (TME), was also higher in the high-risk group than in the low-risk group.

Our results showed that the high-risk group was characterized by immune activation and immune cell infiltration, classified as an immune-inflamed phenotype, and the low-risk group was characterized by the suppression of immunity, classified as an immune-desert phenotype. The immune-inflamed phenotype, known as hot tumor, is sensitive to immunotherapy, while the immune-desert phenotype, known as cold tumor, is insensitive to immunotherapy, revealing that the abundance of immune cell infiltration in the TME is closely linked to immunotherapy efficacy (33). To date, cervical cancer immunotherapy has mainly included ICIs, HPV-related vaccines, dendritic cell-based immunotherapy, and adoptive T cell immunotherapy (34). ICIs have demonstrated marked clinical effects worldwide, and the FDA has approved pembrolizumab for PD-L1 positive metastatic or recurrent cervical cancer (35). To predict the immunotherapy response, we detected the differential expression of checkpoint genes in the low- and high-risk groups. The high-risk group showed a notably high expression of checkpoint genes and was likely to respond well to ICIs. Thus, the lymph node metastasis-related predictive signature could be a reliable tool to evaluate immune cell infiltration in the TME and predict the clinical response to ICI treatment.

Although chemotherapy is a multimodal approach for the treatment of cervical cancer, responses to chemotherapy are limited (36). To identify the most effective antineoplastic agents in the high- and low-risk groups of patients with cervical cancer, we analyzed the IC50 values of 138 chemotherapeutic drugs using the GDSC database. Drug sensitivity analysis showed that docetaxel, paclitaxel, etoposide, erlotinib, lapatinib, sunitinib, gefitinib, and vinorelbine had stronger effects in the low-risk group, whereas cytarabine, PAC-1, and FH535 had stronger effects in the high-risk group. Docetaxel and paclitaxel are commonly used to treat cervical cancer. Both could promote tubulin assembly in microtubules, stabilize microtubules, and inhibit depolymerization to free tubulin, thus blocking cells in the M phase of the cell cycle, representing a class of antineoplastic agents (37). Erlotinib, lapatinib, sunitinib, and gefitinib are tyrosine kinase inhibitors, each of which inhibits cell proliferation and angiogenesis, and their therapeutic effects in advanced and recurrent cervical cancer are still in phase II/III clinical trials (38). Cytarabine is a nucleoside analog that affects cell division in the S phase and acts primarily through inhibition of DNA polymerase; it is mainly used to treat acute myeloid leukemia (39). PAC-1 is the first FDA-approved orphan drug, and its synthetic derivative WF-208 showed fascinating caspase-3-mediated anticancer activity (40). FH535 is a small molecule and dual inhibitor of β-catenin/TCF and PPARs, which has been demonstrated to selectively inhibit the proliferation of pancreatic, hepatocellular, breast, and colorectal carcinoma cells (41). According to current research, cytarabine, PAC-1, and FH535 are less commonly used for the treatment of cervical cancer. However, according to our results, they could also be effective in high-risk lymph node metastasis-related predictive signatures in cervical cancer.

Our study has some limitations. There is still a lack of large-scale clinical trials on *TEKT2* and *RPGR* in cervical cancer with lymph node metastasis, which will be demonstrated in future studies. In addition, we only focused on mRNAs in this study. Several studies have found that non-coding RNAs, such as miRNAs, lncRNAs, and circRNAs, also have significant correlations with lymph node metastasis, which should be considered in further studies.

In summary, we constructed a lymph node metastasis-related predictive signature based on *TEKT2* and *RPGR* expression to predict the prognosis, immune infiltration, and efficiency of individualized therapeutic agents. Our findings not only enrich our understanding of immunotherapy and chemotherapy response in patients with cervical cancer but also provide a new strategy for combining risk stratification with precision treatment of cervical cancer, which might provide novel insights for clinicians.

## Data availability statement

The original contributions presented in the study are included in the article/**Supplementary Material**. Further inquiries can be directed to the corresponding authors.

## Ethics statement

The studies involving human participants were reviewed and approved by the Ethics Committee of the Second Affiliated Hospital of Zhengzhou University (2021040). The patients/participants provided their written informed consent to participate in this study.

## Author contributions

YG, LW, ZX and HZ designed the research. YG, LW, ML, XX and YB performed data collection and analysis. RL, WW, ML and ZX wrote the manuscript draft. YG, ML, XX, RL and HZ prepared the manuscript. All authors contributed to the article and approved the submitted version.

## Supplementary material

Supplementary Figure 1 Establishment of nomogram for survival prediction. **(A)** Nomogram of risk score in lymph node metastasis-related predictive signature. **(B)** Calibration curve for 1-year survival of the nomogram. **(C)** Calibration curve for 3-years survival of the nomogram. **(D)** Calibration curve for 5-years survival of the nomogram. **(E)** ROC curve for 1-, 3-, and 5-years survival of the nomogram.

Supplementary Figure 2 Functional enrichment analysis in lymph node metastasis-related predictive signature. **(A)** GO functional analysis between high- and low-risk groups in cervical cancer. **(B)** KEGG functional analysis between high- and low-risk groups in cervical cancer. **(C)** Correlation analysis between KEGG pathways and the expression of risk score, *TEKT2* and *RPGR*. Blue represents negative correlation, and red represents positive correlation.

Supplementary Figure 3 Assessment of immune microenvironment in lymph node metastasis-related predictive signature. **(A)** Correlation analysis between immune cells and the expression of *TEKT2* and *RPGR*. Blue represents negative correlation, and red represents positive correlation. **(B)** Correlation analysis between checkpoint genes and the expression of *TEKT2* and *RPGR*. Blue represents negative correlation, and red represents positive correlation. **(C)** Differential expression analysis of HLA family genes in high- and low-risk groups. The asterisks represented the statistical p value (**P* < 0.05; ***P* < 0.01).
