# Supplementary material for: Lymph node metastasis-related gene signature shows good performance in predicting prognosis and immune infiltration in cervical cancer
Source: Front Oncol. 2023 Jun 22;13:1190251. doi: 10.3389/fonc.2023.1190251 (PMC10325684; doi:10.3389/fonc.2023.1190251)
Supplement: Supplementary file 1 [file Table_1.docx]

**Supplementary Table1:** Primer Sequences for qRT-PCR

| **Gene** | **Primer Sequences (5–3)** |
| --- | --- |
| **GAPDH** |  |
| Forward | GGAAGCTTGTCATCAATGGAAATC |
| Reverse | TGATGACCCTTTTGGCTCCC |
| **TEKT2** |  |
| Forward | GAAGGACCCTGTGGAGGATGA |
| Reverse | ACTTCCTGCAAGAGGCAGAGC |
| **RPGR** |  |
| Forward | TACATCCGAGCATAAGATTAAGCAG |
| Reverse | GCTGAGGGACACAGACATTACTTAC |
