# Supplementary material for: Lymph node metastasis-related gene signature shows good performance in predicting prognosis and immune infiltration in cervical cancer
Source: Front Oncol. 2023 Jun 22;13:1190251. doi: 10.3389/fonc.2023.1190251 (PMC10325684; doi:10.3389/fonc.2023.1190251)
Supplement: Supplementary file 2 [file Table_2.docx]

**Supplementary Table2:** The siRNA sequences of TEKT2

| **Gene** | **Primer Sequences (5–3)** |
| --- | --- |
| **siRNA-1** |  |
| Sense | GACCAACAACGAGCUUGAA(dT)(dT) |
| Antisense | UUCAAGCUCGUUGUUGGUC(dT)(dT) |
| **siRNA-2** |  |
| Sense | GACAAGUGUCUGACAGAUU(dT)(dT) |
| Antisense | AAUCUGUCAGACACUUGUC(dT)(dT) |
| **siRNA-3** |  |
| Sense | CCUCUUGCAGGAAGUCCAA(dT)(dT) |
| Antisense | UUGGACUUCCUGCAAGAGG(dT)(dT) |
