# Supplementary figures and images for: Lymph node metastasis-related gene signature shows good performance in predicting prognosis and immune infiltration in cervical cancer
Source: Front Oncol. 2023 Jun 22;13:1190251. doi: 10.3389/fonc.2023.1190251 (PMC10325684; doi:10.3389/fonc.2023.1190251)

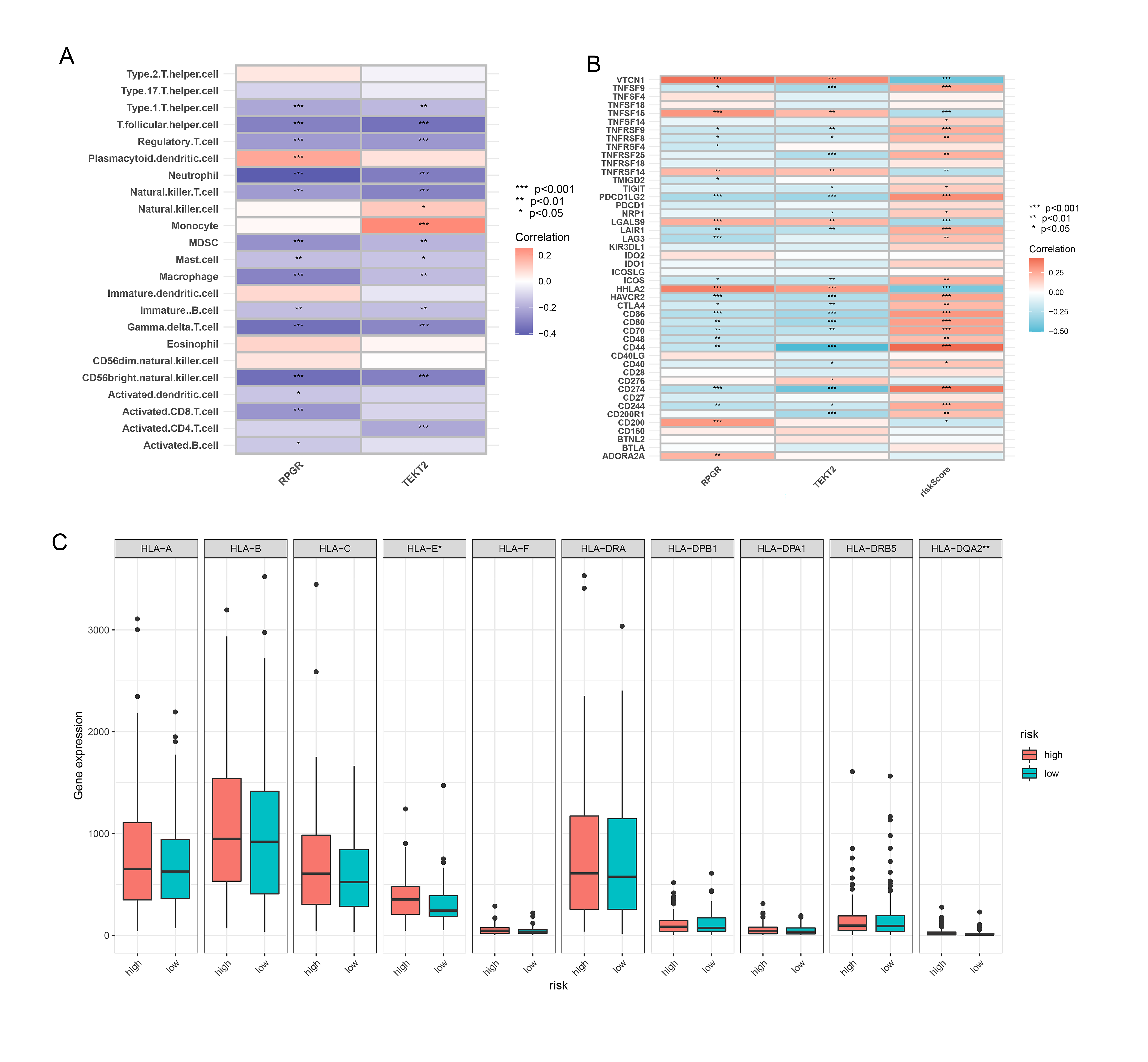

Supplement: Supplementary Figure 3 — Assessment of immune microenvironment in lymph node metastasis-related predictive signature. (A) Correlation analysis between immune cells and the expression of TEKT2 and RPGR. Blue represents negative correlation, and red represents positive correlation. (B) Correlation analysis between checkpoint genes and the expression of TEKT2 and RPGR. Blue represents negative correlation, and red represents positive correlation. (C) Differential expression analysis of HLA family genes in high- and low-risk groups. The asterisks represented the statistical p value (*P < 0.05; **P < 0.01). [file Image_3.tif]
